# Supplementary material for: Overexpression of a Grape MYB Transcription Factor Gene VhMYB2 Increases Salinity and Drought Tolerance in Arabidopsis thaliana
Source: Int J Mol Sci. 2023 Jun 28;24(13):10743. doi: 10.3390/ijms241310743 (PMC10341777; doi:10.3390/ijms241310743)
Supplement: Supplementary file 1 [file ijms-24-10743-s001.zip › Supplementary Figure S1.pdf]

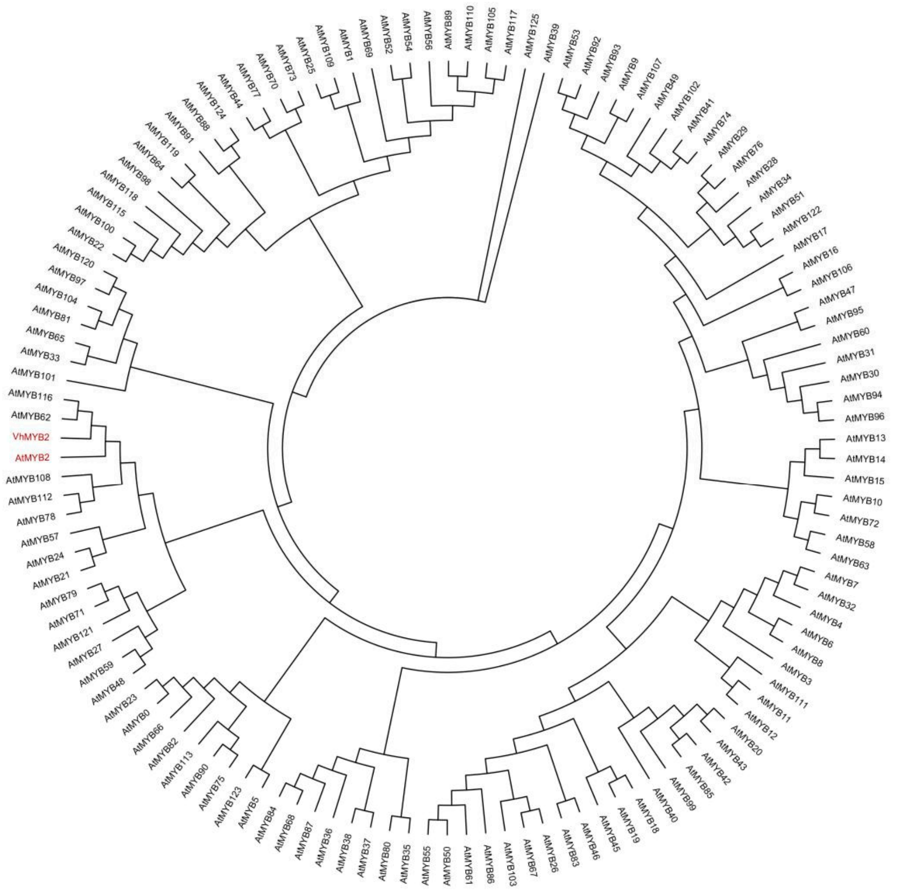

Figure S1. Phylogenetic tree of VhMYB2 protein and all AtMYB proteins. The red fonts indicate the VhMYB2 and AtMYB2 proteins.
