# Supplementary material for: Overexpression of a Grape MYB Transcription Factor Gene VhMYB2 Increases Salinity and Drought Tolerance in Arabidopsis thaliana
Source: Int J Mol Sci. 2023 Jun 28;24(13):10743. doi: 10.3390/ijms241310743 (PMC10341777; doi:10.3390/ijms241310743)
Supplement: Supplementary file 1 [file ijms-24-10743-s001.zip › Supplementary Table S1.pdf]

Table S1. List of primers used in this study.

**Primer Name**	**Primer Sequence (5'→3')**	**Purpose**
VhMYB2-qF	AGAGTTGCAGGCTGAGATGGTT	qPCR
VhMYB2-qR	CAGGTGTTGGGCTATTTTGGA	qPCR
VvActin-F	CTTGCATCCCTCAGCACCTT	qPCR
VvActin-R	TCCTGTGGACAATGGATGGA	qPCR
VhMYB2-F	ATGGTGGTGAGCGAGAGAGG	full-length cDNA of *VvMYB2*
VhMYB2-R	CTAGAAGTCGTCAAAGAACTGTTGC	full-length cDNA of *VvMYB2*
HR-F	ATTTGGAGAGGACAGGGTACCATGGTGGTGAGCGAGAGAGG	PCR for homologous recombination
HR-R	TGCCTGCAGGTCGACTCTAGAGAAGTCGTCAAAGAACTGTTGCCTT	PCR for homologous recombination
VhMYB2-slF	GAGCTCGGTACCCGGGGATCCATGGTGGTGAGCGAGAGAGG	For subcellular location
VhMYB2-slR	GCCCTTGCTCACCATGTCGACGAAGTCGTCAAAGAACTGTTGCCTT	For subcellular location
AtP5CS1-F	AGGGAAAGTTCCAGAAAG	qPCR
AtP5CS1-R	CATAACTAAGCGAGCCAC	qPCR
AtCAT1-F	GTCCTGGGATTCAGACAGGC	qPCR
AtCAT1-R	GGCCTCACGTTAAGACGAGT	qPCR
AtNCED3-F	TTGATGCTCCAGATTGCTTC	qPCR
AtNCED3-R	GGACCCTATCACGACGACTT	qPCR
AtSnRK2.6-F	AGATCCCGAGGAACCAAAGA	qPCR
AtSnRK2.6-R	CTCTTTGCAGGGTCAGCAAC	qPCR
AtSOS2-F	GCAAGGGAAGAAGAAGAAGT	qPCR
AtSOS2-R	TCTCCGCTACATAACTGCC	qPCR
AtSOS3-F	GAATCCATCGCTCATCAA	qPCR
AtSOS3-R	CCATTTCTTCCTCTTCACA	qPCR

AtSOS1-F	TTCATCATCCTCACAATGGCTCTAA	qPCR
AtSOS1-R	CCCTCATCAAGCATCTCCCAGTA	qPCR
AtNHX1-F	AGCCTTCAGGGAACCACAAT	qPCR
AtNHX1-R	CTCCAAAGACGGGTCGCATG	qPCR
AtActin-F	TTACCCGATGGGCAAGTCA	qPCR
AtActin-R	AAACGAGGGCTGGAACAAGA	qPCR
